# Stability of Plant Leaf-Derived Extracellular Vesicles According to Preservative and Storage Temperature

**DOI:** 10.3390/pharmaceutics14020457

**Published:** 2022-02-21

**Authors:** Kimin Kim, Jungjae Park, Yehjoo Sohn, Chan-Eui Oh, Ji-Ho Park, Jong-Min Yuk, Ju-Hun Yeon

**Affiliations:** 1 Department of Integrative Biosciences, University of Brain Education, Cheonan 31228, Korea; kimini1127@naver.com (K.K.); spielian@naver.com (Y.S.); 97rhdaud@naver.com (C.-E.O.); 2 Department of Materials Science and Engineering, Korea Advanced Institute of Science and Technology, Daejeon 34141, Korea; jungjae10@kaist.ac.kr (J.P.); jongmin.yuk@kaist.ac.kr (J.-M.Y.); 3 Department of Bio and Brain engineering, Korea Advanced Institute of Science and Technology, Daejeon 34141, Korea; jihopark@kaist.ac.kr

**Keywords:** plant-derived extracellular vesicles, stability, preservative, freeze-thawing cycles

## Abstract

Plant-derived extracellular vesicles (EVs) are capable of efficiency delivering mRNAs, miRNAs, bioactive lipids, and proteins to mammalian cells. Plant-derived EVs critically contribute to the ability of plants to defend against pathogen attacks at the plant cell surface. They also represent a novel candidate natural substance that shows potential to be developed for food, cosmetic, and pharmaceutical products. However, although plant-derived EVs are acknowledged as having potential for various industrial applications, little is known about how their stability is affected by storage conditions. In this study, we evaluated the stability of *Dendropanax morbifera* leaf-derived extracellular vesicles (LEVs) alone or combined with the preservatives, 1,3-butylene glycol (to yield LEVs-1,3-BG) or TMO (LEVs-TMO). We stored these formulations at −20, 4, 25, and 45 °C for up to 4 weeks, and compared the stability of fresh and stored LEVs. We also assessed the effect of freeze-thawing cycles on the quantity and morphology of the LEVs. We found that different storage temperatures and number of freeze-thawing cycles altered the stability, size distribution, protein content, surface charge, and cellular uptake of LEVs compared to those of freshly isolated LEVs. LEVs-TMO showed higher stability when stored at 4 °C, compared to LEVs and LEVs-1,3-BG. Our study provides comprehensive information on how storage conditions affect LEVs and suggests that the potential industrial applications of plant-derived EVs may be broadened by the use of preservatives.

## 1. Introduction

Extracellular vesicles (EVs) contain DNA, RNA, proteins, and lipids. EVs mediate cell-to-cell communication by delivering a variety of molecules, and allow cross-kingdom communication between plants and animals [1,2]. Plant-derived EVs are structurally similar to mammalian exosomes [3]. They successfully mediate bioactive components intercellular communication, owing to their small nano-sized EVs [4]. These characteristics suggest that EVs could potentially be developed for applications in the cosmetic and food industries [5].

Recent studies on food-derived EVs have shown that these EVs are safe, non-toxic to humans, and even modulate cellular processes involved in health and disease [6,7]. In addition, there is a report that exosomes perform biological functions in the skin [8]. Kim et al. suggested an important role of stem cell EVs in the initiation and progression of skin aging [9]. Cho et al. showed plant exosomes have the potential to be commercialized as a cosmeceutical product [10,11]. Despite the high potential for using EVs in the cosmetic and food industries, little effort has been made to standardize or optimize their storage conditions, especially for plant-derived exosomes [12].

Exosomes are most often used when freshly isolated, but it has been reported that they can be stored at −80 °C for up to 1 year [12,13,14]. However, repeated freeze-thaw cycles can structurally distort the membrane and impact the shelf life of exosomes, and the physicochemical properties of exosomes can be affected by a variety of other storage conditions, including pressure, the properties of the utilized solvent(s), and the storage period. Cosmetic raw materials need to be able to withstand extreme temperature changes during transportation, and freeze-thaw testing is highly useful for liquid-based cosmetics [15]. Given the above, exosome preservation methods should be optimized to protect their biological activities and increase the convenience of their transportation and industrial application [13].

The current methods for storing EVs mainly include freezing, freeze-drying, and spray-drying [13]. Freezing is a widely accepted storage method that uses cryoprotectants to maintain protein stability [16]. In freeze-drying (or lyophilization), a moisture-containing material is frozen such that any ice is sublimated to water vapor. In spray-drying [17], the solution is atomized and hot air is used to quickly convert it into a dry powder [16,18].

We should understand the relationship between shelf life and integrity of EVs if we hope to efficiently apply EVs for commercial production [14]. The conditions for preserving and storing EVs must be optimized to ensure their stability [19]. A preservative is a natural or synthetic compound added to a pharmaceutical or cosmetic preparation to avert spoilage due to microbial growth [20]. Natural preservatives could be an alternative to synthetic preservatives in the cosmetic and food industries [21]. The 1,3-BG (1,3-butylene glycol) as a natural preservative is a highly viscous, colorless, odorless, and transparent liquid with low volatility and low toxicity for people of most skin types [22]. It has been used in cosmetic products at concentrations of 0.1–50% as a moisturizer, anti-microbial, and solvent for plant extracts and fragrances [23,24]. Saliguard TMO is a preservative that has been developed as an alternative to parabens and formaldehyde-free preservatives for the cosmetic industry. TMO consists of an extract of *Illicium verum*, caprylyl glycol, 1,2-hexanediol, and butylene glycol. *Illicium verum* extract has been widely used in traditional medicine as a bioactive compound and a pharmaceutical treatment for many diseases [25,26]. It shows anti-oxidant, preservative, and antimicrobial properties, and a number of its oil constituents induce synergistic biocidal effects against pathogenic fungi and mycotoxin production [27,28]. Caprylyl glycol is found in a wide variety of preservatives with broad-spectrum antimicrobial properties; it acts to disrupt the microbial cell membrane [29,30]. 1,2-Hexanediol has been widely used in the cosmetic industry as a preservative with antimicrobial activity. It exhibits broad-spectrum activity against Gram-positive and Gram-negative bacteria, potentially by binding to and altering the cytoplasmic membrane, leading to its rupture [31,32].

*Dendropanax morbifera*, which is endemic to southern parts of Asia, has historically been used in traditional medicine and the commercial production of golden varnish. *Dendropanax morbifera* extracts have been shown to have anticancer [33,34], antioxidant [35], antidiabetic [36], anti-inflammatory [37], and anti-melanogenic effects [5]. We previously developed a technology to isolate plant-derived EVs and used it to isolate EVs from *Dendropanax morbifera* [5]. We found that leaf-derived extracellular vesicles (LEVs) from *Dendropanax morbifera* had anti-melanogenic effects [5]. However, little is known about the optimal storage conditions of LEVs with and without preservatives.

In this study, we investigated the physical stability of *Dendropanax morbifera* LEVs alone and when combined with the preservatives, 1,3-BG (LEVs-1,3-BG) or TMO (LEVs-TMO). The preservatives, which met the criteria of being tasteless, odorless, colorless, non-irritating, and antimicrobial [38], were intended to inhibit the growth of microorganisms and needed to last longer than the cosmetic and food product itself. In addition to time, temperature and pH play important roles in modulating the physical stability of EVs, such as by provoking their decomposition or modifying their preservative activity. Furthermore, we monitored EV size and protein levels over time and at different temperatures.

We found that LEVs were stable when incubated at 4 °C for 0 to 2 weeks, as measured by DLS (dynamic light scattering) and protein level analysis. LEVs-TMO exhibited more homogeneous-sized particles compared with LEVs and LEVs-1,3-BG. Furthermore, LEVs-TMO were stable at 4 °C for an extended period. Thus, the storage of naturally generated EVs under an improved preservation condition may support a new direction for delivering for nutraceutical or cosmeceutical compounds into cells. Collectively, our findings suggest that using favorable preservation conditions may facilitate the development of natural plant-derived EVs for industrial applications. This technique could be a new strategy with multiple purposes for commercial use in the exosomes-based food raw materials without preservation that can be used for a short term, as well as exosomes-based cosmetic market from natural plants in the future, when combined with TMO preservative.

## 2. Materials and Methods

### 2.1. Isolation of D. morbifera Leaf-Derived Extracellular Vesicles

We collected fresh leaves of *Dendropanax morbifera* from Bogil Island, which is located in Wando-gun, Jeollanam-do, South Korea. Unlike the general method for isolating plant vesicles [39,40,41], we have developed a method for isolating EVs from *Dendropanax morbifera* leaf to facilitate industrial application. *Dendropanax morbifera*-derived EVs were isolated by processing the leaves with a mixer grinder plus extractor, passing the resulting crude leaf extract through filter paper, and centrifuging the obtained extract at 10,000× *g* for 10 min. Then, large debris was removed by filtering the supernatant through a 0.22-μm membrane, and then the filtered EVs were concentrated by centrifuging the sample at 5000× *g* for 10 min at 4 °C in an Amicon Ultra-4 PL 100 K concentrator (Merck Millipore, Darmstadt, Germany) [5]. After isolating LEVs, we measured the protein concentration using a Pierce bicinchoninic acid (BCA) protein assay kit (Thermo Fisher Scientific, Waltham, MA, USA), and prepared it by dilution with distilled water to calculate equal concentration of LEVs with and without preservatives. After centrifugation, the protein concentration of EVs was estimated using a Pierce bicinchoninic acid (BCA) protein assay kit (Thermo Fisher Scientific, Waltham, MA, USA).

### 2.2. Combining LEVs with Preservatives

To test how preservatives affected the storage behavior of LEVs, we added 1,3-BG or TMO as natural preservative into the crude leaf extract used for isolation of the LEVs. Theses preservatives have been used in cosmetic products at concentrations ranging from 0.1 to 50% as moisturizers, anti-microbials, and solvents for plant extracts and fragrances. Natural preservatives have been extensively explored for their antioxidant and antimicrobial properties in food preservation systems. The LEVs were mixed with 1,3-BG at a ratio of 7:3 (*w*/*w*) to generate LEVs-1,3-BG or with TMO at a ratio of 99.5:0.5 (*w*/*w*) to generate LEVs-TMO. The LEVs-TMO consisted of LEVs (3.3%), *Illicium verum* (anise) fruit extract (0.05%), caprylyl glycol (0.15%), 1,2-hexanediol (0.2%), butylene glycol (0.1%), and water (96.2%).

### 2.3. Evaluation of Physical Stability

The physical stabilities of LEVs, LEVs-1,3-BG, and LEVs-TMO were evaluated over 4 weeks under storage at −20, 4, 25, and 45 °C. Physical properties were evaluated at the beginning of the experimental period (week 0) and after 1, 2, 3, and 4 weeks of storage. The parameters assessed were odor, color, phase separation, and pH.

### 2.4. Size Characterization of Stored LEVs under Different Preservative and Temperature Conditions

Dynamic light scattering (DLS) was used to assess the hydrodynamic size distribution profiles of the various formulations, as applied with a Zetasizer nano ZS90 system (Malvern Panalytical, Malvern, UK). Collected LEVs were placed in a thermostatic cell at 20 °C, and measurements were obtained using the scattered intensity autocorrelation function. For zeta potential measurement, the LEVs were mixed with distilled water at a ratio of 95:3 (*v*/*v*). Each diluted sample was inserted into a folded capillary cell (DTS1070; Malvern Instruments) and detected using a Zetasizer nano ZS90 system.

### 2.5. Protein Quantification

The concentration of proteins associated with LEVs of the different formulations was assessed using a Pierce BCA assay kit. A standard curve was prepared by mixing 10 μL of BCA standard solution and 200 μL of BCA working solution in a 96-well plate, and incubating the mixture at 37 °C for 30 min. Absorbance was evaluated at 562 nm using a microplate reader (BioTek, Winooski, VT, USA) under storage at −20, 4, 25, and 45 °C. Protein levels were evaluated during 4 weeks of storage.

### 2.6. Stability Testing under Freeze-Thaw Cycles

Samples were subjected to zero, one, or three freeze-thaw cycles consisting of −20 °C for 24 h followed by a return to room temperature (23 °C) for 24 h. After each cycle, the size distribution was determined using DLS.

### 2.7. Transmission Electron Microscopy (TEM)

For TEM analysis, 4 μL of a sample solution consisting of LEVs or LEV-TMO was loaded onto Cu 200-mesh carbon film grid (Electron Microscopy Science, PA, USA) that had been surface-treated with glow discharge. The grid was incubated with the sample for 1 min, washed three times with 20 μL of distilled water, and stained with 100 μL of 2% (*w*/*v*) uranyl acetate solution. Excess staining solution was removed with Whatman filter paper (GE Healthcare Life Science, Buckinghamshire, UK), and the grids were air-dried for 10 min and observed using an JEM-2100F (JEOL Ltd., Tokyo, Japan) equipped with a field emission gun and One View camera (Gatan Inc., CA, USA). An acceleration voltage of 200 kV was used.

### 2.8. Imaging Analysis of Intracellular LEVs and LEVs-TMO Subjected to Freeze-Thaw Cycles

The internalization of LEVs and LEV-TMO was analyzed by fluorescence microscopy. LEVs and LEVs-TMO were incubated with lipophilic Di-I (MOP-D-3911l; Invitrogen, Waltham, MA, USA) for 30 min at 37 °C, and transferred to 100 kDa filter to removed free Di-I dye. We previously confirmed the anti-melanogenic effects and monitored internalization of LEVs into B16BL6 murine melanoma cells [5]. B16BL6 melanoma cells were cultured in α-minimum essential media (α-MEM) (Gibco, Thermo Fisher Scientific, Waltham, MA, USA) supplemented with 10% foetal bovine serum (Rocky Mountain Biologicals, Missoula, MT, USA), and 1% penicillin/streptomycin (Lonza, Basel, Switzerland). The cells were incubated at 37 °C in a humidified 5% CO_2_ atmosphere. To investigate whether preservatives affect the internalization of LEVs, B16BL6 were treated with tagged 1 mg/mL LEVs and LEVs-TMO for 3 h, the medium was removed, and cells were washed three times with PBS and fixed with 4% paraformaldehyde. Hoechst 33342 (Invitrogen, Carlsbad, CA, USA) was added and the cells were incubated at room temperature for 15 min to stain nuclei. Finally, the cells were washed with 1% bovine serum albumin (BSA) and imaged under a fluorescence microscope (Leica Microsystem, Wetzlar, Germany). At least three images were analyzed per sample using the Image J software (U.S. National Institutes of Health, Bethesda, MD, USA).

## 3. Results and Discussion

### 3.1. Physical Stability of LEVs, LEV-1,3-BG, and LEVs-TMO

To assess the effect of preservatives, we mixed the LEVs with 1,3-BG or TMO. We then measured the odor, color, phase separation, pH, and size distribution of the LEVs (taken as reflecting their stability) at −20, 4, 25, and 45 °C. Measurements were performed at baseline and weekly thereafter for 4 weeks. The results obtained for these assessments are presented in Tables S1–S3. We did not observe any change in the color (light yellow) or odor (similar to that of the raw materials), nor was there any apparent phase separation during 4 weeks of storage at 4, 25, or 45 °C. In contrast, LEVs-1,3-BG exhibited phase separation, as evidenced by our observation of soluble sediment, which is dissolved by shaking accumulating sediment from week 1 to 4 (1w–4w) of storage at 45 °C. This was verified under optical microscopy (Figure S1). We observed phase separation in LEVs-1,3-BG compared to LEVs and LEVs-TMO, which is transparent under microscopy.

**Figure 1:**
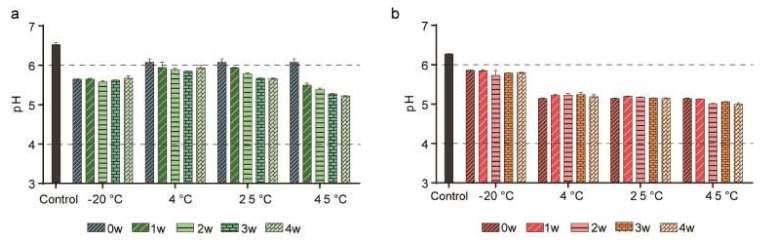
pH values for LEVs stored with and without preservatives at different temperatures. pH values for (**a**) LEVs and (**b**) LEVs-TMO following storage for 4 weeks at −20, 4, 25, and 45 °C. Data are presented as mean ± standard error of mean (SEM).

Figure 1 showed the average pH of the stored formulations over time. The average pH of LEVs and LEVs-1,3-BG were about pH 6 at 0w, whereas that of LEVs-TMO was about pH 5 in Figure 1 and Figure S2. The pH of LEVs and LEVs-1,3-BG tended to decrease over time, although the total change was relatively minor (<1 unit) over 4 weeks. In contrast, the pH of LEVs-TMO was stable and remained about pH 5 throughout the experimental period in all storage conditions. As shown in Figure 1, the pH change of LEVs stored for 4 weeks under various temperatures could be relatively minimal reduced by the addition of TMO.

As skin pH values range from 5.0 and 6.0 [42], LEVs with and without preservatives had pH values within the average pH range of the skin. Stability testing of cosmetic products is used to predict the physical and chemical changes that may occur during their shelf life. Such testing seeks to provide information on how instability could manifest, and to suggest possible changes that could be made to the product before it is released [43]. We could predict stability of LEVs by combining preservatives throughout trends of pH during 4 weeks.

### 3.2. Physiological Properties of LEVs, LEV-1,3-BG, and LEVs-TMO over Storage Time and Under Different Temperatures

EVs can be characterized according to their physical properties, surface charge, and protein concentration [44]. The particle size of LEVs is considered to be a key element, because many properties of nanomaterials depend on their size [33,45].

**Figure 2:**
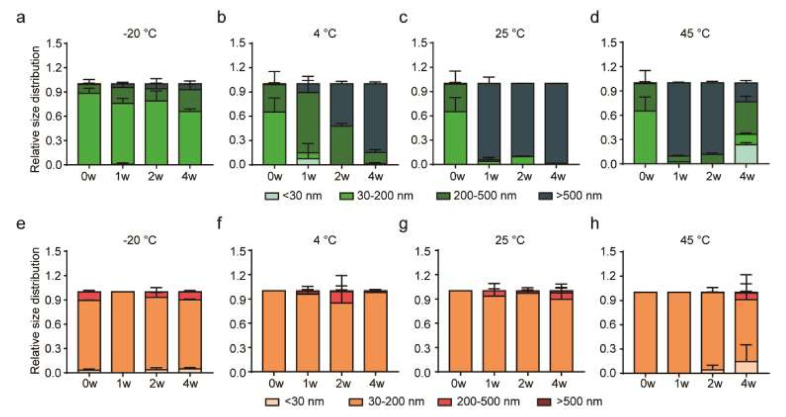
Size distribution changes over time in the various formulations stored at different temperatures. (**a**–**d**) Size distributions over time for LEVs stored at −20, 4, 25, and 45 °C. (**e**–**h**) Size distributions over time for LEVs-TMO stored at −20, 4, 25, and 45 °C. Data are presented as mean ± standard error of mean (SEM).

To investigate the influence of different storage temperatures and preservatives, we measured the size range of LEVs, LEVs-1,3-BG, and LEVs-TMO at −20, 4, 25, and 45 °C. For LEVs (Figure 2a–d), the particle diameters ranged mainly between 30 and 200 nm at 0w, and polydispersity index (PDI) values were approximately 0.2–0.3 (Figure S3). The diameters of those stored at −20 °C were mainly within the 30–200 nm range over the 4-week period, but the proportion of particles with diameters 200–500 nm increased over time. The diameter of LEVs stored at 4 °C were increased sizes larger than 500 nm by DLS over time, and the diameter of LEVs stored at 25, 45 °C were mainly sizes larger than 500 nm from 2 weeks in Figure 2a–c.

At 0w, the particles of LEVs-1,3-BG were generally smaller than 30 nm (Figure S4a–c), indicating that the LEVs were affected by the presence of 1,3-BG. The proportions of LEVs-1,3-BG particles larger than 500 nm increased over time under storage at −20, 4, 25, and 45 °C. Optical microscopy revealed the presence of a soluble sediment beginning at 2w in samples stored at 45 °C (Figure S1). In particular, the average 10-µm-sized particles of LEVs-1,3-BG were increased from 2 weeks at 45 °C. Based on these results, we speculate that LEVs mixed with 1,3-BG may fuse into larger vesicles depending on the temperature.

Based on these results, we found that LEVs-TMO was more stable in physiological properties compared to LEVs-1,3-BG. We selected LEVs-TMO for stability studies. The zeta potential of an EV is its surface charge, and can be estimated from electrophoretic mobility within colloidal dispersion [46]. We measured the zeta potentials of LEVs and LEVs-TMO at −20, 4, 25, and 45 °C. The results are presented in Figure S5a–d. The zeta potentials of LEVs and LEVs-TMO showed minimal difference when stored at —20 °C, and there was not significant difference when stored at 4, 25 °C from 0 to 4w (Figure S5a–c). That of LEVs-TMO stored at 45 °C was changed to negative charge at 4w (Figure S5d). Zeta potentials may have functional consequences for the EVs, such as by inducing alterations in aggregation, protein structures, surface modifications, and/or protein unfolding/denaturation. As Maroto et al. reported that freezing of exosomes can diminish their zeta potential [40], we found that the surface charge of LEVs-TMO was slightly decreased at −20 °C.

**Figure 3:**
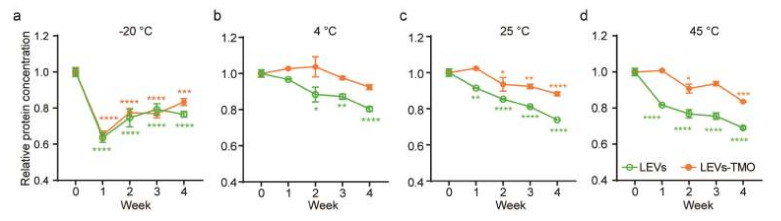
Total protein concentrations associated with LEVs stored with and without preservatives at different temperatures. The relative protein concentrations of LEVs and LEVs-TMO stored at (**a**) −20, (**b**) 4, (**c**) 25, and (**d**) 45 °C for 4 weeks were detected by BCA. Data are presented as mean ± standard error of mean (SEM) (* *p* < 0.05, ** *p* < 0.01, *** *p* < 0.001, **** *p* < 0.0001).

To examine quantity change during storage, we measured the total protein level under different temperatures and increasing storage time. We used the bicinchoninic acid (BCA) assay to monitor the protein levels present in equal amounts of LEVs and LEVs-TMO (Figure 3a–d). The protein levels of LEVs and LEVs-TMO showed a general decrease of protein level from 0 to 4 weeks. However, decreasing trends of protein levels in LEVs were more rapid than in LEVs-TMO. In particular, LEVs and LEVs-TMO stored at −20 °C exhibited rapid decreases in protein levels beginning at 1 w (Figure 3a). This suggests that the protein content of these EVs could be decreased by the freezing process. Cheng et al. reported that the concentration of exosomal proteins was decreased the most by storage at −20 °C, compared to the other tested temperatures, as assessed by nanoparticle tracking analysis (NTA) [47]. These results may indicate that freezing leads to a loss of exosomal contents. Notably, although we found that the total protein level decreased for both LEVs and LEVs-TMO stored at 25 or 45 °C (Figure 3b,c), the highest protein level was maintained over 4 weeks for LEVs-TMO stored at 4 °C (Figure 3a).

Sokolova et al. found that exosomes exhibited a smaller size change at 37 °C compared to 4 °C [48]. Another study showed that animal EVs stored at 4 ℃ had the highest exosome concentration and higher levels of the representative exosome markers, ALIX, HSP70, and TSG101 [47]. Higher temperatures were found to be unsuitable for storage of exosomes due to degradation of exosomal proteins [12]. Here, we found that the particle sizes of LEVs came to range widely (from 10 to 10,000 nm) when LEVs were stored for 4 weeks at −20, 4, 25, and 45 °C, whereas LEVs-TMO remained largely in the range of 30–200 nm throughout the experimental period, and thus resembled the control value at day 0. Our results support the idea that selecting an appropriate preservation method for LEVs could support function of extracellular vesicles and thereby make LEVs more useful for industrial applications.

### 3.3. Physiological Properties of LEVs and LEV-TMO under Various Numbers of Freeze-Thaw Cycles

**Figure 4:**
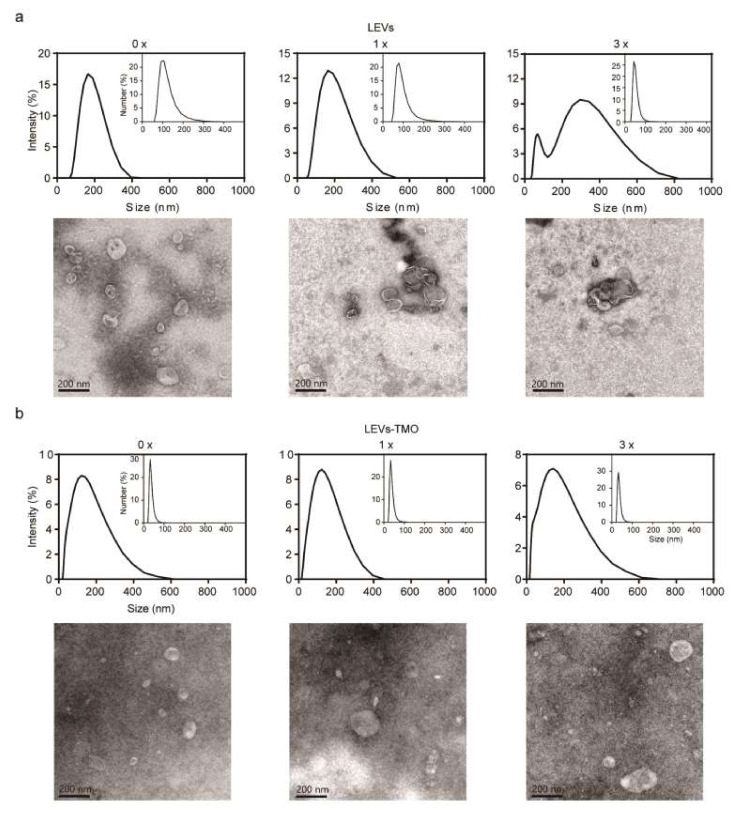
Size distributions of LEVs and LEVs-TMO over freeze-thaw cycles. (**a**) Top: Dynamic light scattering (DLS) measurements of the size distribution intensity for LEVs exposed to 0, 1, and 3 freeze-thaw cycles. Insets: number-size distribution curves. Bottom: TEM images of LEVs exposed to different numbers of freeze-thaw cycles. (**b**) Top: DLS measurements of the size distribution intensity for LEVs-TMO exposed to 0, 1, and 3 freeze-thaw cycles. Insets: number size distribution curves. Bottom: TEM images of LEVs-TMO of different freezing and thawing cycles. Data are presented as mean ± standard error of mean (SEM).

It is currently recommended that EVs be maintained at −80 °C for transportation and storage. However, this temperature may alter the biological activities and/or morphological characteristics of EVs. In addition, it is expensive to transport materials at −80 °C [17,49,50]. Furthermore, freeze-thaw may encounter during transportation. We should investigate whether the raw materials remain stable. To evaluate the effect of freeze-thaw cycles, LEVs and LEVs-TMO were frozen to −20 °C and thawed to room temperature for 1 or 3 cycles (Figure 4) and analyzed by DLS and TEM. Prior to freeze-thawing, LEVs were relatively spherical nanoparticles with a diameter of approximately 100–150 nm (Figure 4a). However, with the increasing cycles of freezing and thawing, LEVs were larger than 200 nm and a size distribution histogram showed a wider than 0-cycle of LEVs, as measured DLS, which aggregated and clogged LEVs, shown in Figure 4a. The results of our TEM analyses were consistent with those obtained from the DLS measurements. TEM analyses show that LEVs exhibited a spherical morphology at cycle 0 and then, aggregated or disrupted at increased cycles of freeze and thaw. In addition, TEM images show that LEVs-TMO exhibited a spherical morphology at 0–3 cycles but seem to expand in size with increasing freeze-thaw cycles.

Some reports described that size changes and heterogeneous shapes were observed upon thawing of frozen exosome samples, and suggested that freezing without cryopreservation may affect the stability of exosome membranes during thawing [51,52]. The hydrodynamic diameters of LEVs-TMO were approximately 100 nm and small nano vesicles of approximately 30 nm were observed. The hydrodynamic diameter of LEVs-TMO remained similar to that of 0-cycle LEVs-TMO as the freezing and thawing cycle increased (Figure 4b). This phenomenon may reflect that the preservative (TMO) affected the size of the LEVs, but increased their stability in the face of freeze-thawing.

### 3.4. Freeze-Thawing-Related Differences in the Cellular Uptake of LEVs and LEV-TMO

**Figure 5:**
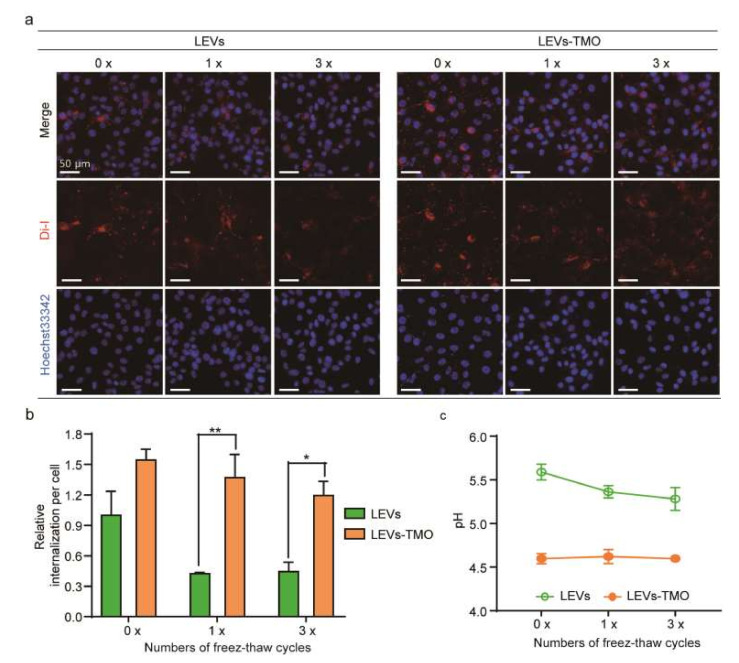
Comparison of cellular uptake for LEVs with and without preservatives. (**a**) Representative fluorescence microscopic images of cellular uptake over 3 h for fluorescently labeled LEVs and LEVs-TMO previously exposed to 0, 1, and 3 freeze-thaw cycles. (**b**) Summary data comparing intracellular fluorescence intensity per cell between LEVs and LEVs-TMO treated as described in (**a**). * *p* < 0.05, ** *p* < 0.01. (**c**) pH values for stored solutions of LEVs and LEVs-TMO exposed to 0, 1, and 3 freeze-thaw cycles.

Storage conditions were also found to influence the cellular uptake of LEVs. To investigate how the different storage conditions impacted the cellular uptake of LEVs, we subjected DiI-labeled LEVs to 0, 1, and 3 cycles of being frozen to −20 °C and thawed to room temperature. We then applied the treated formulations to B16BL6 cells for 3 h and assessed the uptake of the labeled EVs. Our results revealed that LEVs without freeze-thawing were widely distributed within cells, including in the cytoplasm surrounding the nucleus (Figure 5a). As the number of freeze-thaw cycles increased, the cellular uptake efficiency decreased for LEVs (Figure 5b). This may be due to the freeze-thaw-induced aggregation of LEVs observed by TEM (Figure 4), and/or to freeze-thaw-induced damage that is enhanced by repeated cycles [53]. The uptake of the labeled LEVs showed a significant decrease of autologous cellular uptake efficiency under increased cycles of freezing and thawing, but 1, 3 cycles of uptake of the labeled LEV-TMO were less affected compared to 0 cycle in Figure 5b. However, the BCA protein level between LEVs and LEVs-TMO was not significantly decreased by 1 or 3 freeze-thaw cycles (data not shown). In Figure 3a, the LEV and LEV-TMO stored at −20 °C resulted in decreased protein levels at 1 w. The protein contents of short-term storage conditions about freezing-thawing cycles may have less effect than these of long-term storage conditions. LEVs stored at 25 °C showed higher cellular uptake than LEVs stored at −20 °C and 4 °C. On the other hand, we observed that the LEVs stored at 45 °C were difficult to uptake into cells because lipophilic Di-I aggregates at high temperature (Figure S6).

We found that the internalized LEVs were significantly decreased in the cytoplasm, whereas the cellular uptake of similarly treated LEVs-TMO was relatively stable. The size of EVs is an important factor that influences cellular uptake [54]. We found that the size of LEVs-TMO was about 100 nm, which is smaller than that of LEVs. As shown in Figure 4a, LEVs showed agglomeration and aggregation during freeze-thaw cycles on TEM images, which may be one of the reasons for the low cellular uptake. Furthermore, as some reports found that increased exosome uptake occurred at low pH [47,55], we speculate that the increased cellular uptake of LEVs-TMO was due to the decrease in the pH level (Figure 5c). Future studies are warranted to improve our understanding of how pH influences exosomes. Interestingly, the relative number of freeze-thaw cycles was reported to change the membrane properties of exosomes, enabling them to be more easily absorbed by cells [47]. However, our results showed that an increased number of freeze-thaw cycles decreased the cellular uptake of LEVs. Clearly, further biochemical research is needed to clarify the various parameters that are relevant for exosomal uptake.

Our findings indicated that LEVs-TMO were more stable than LEVs or LEVs-1,3-BG, exhibiting smaller changes in pH and size with time and temperature. pH changes may influence the internalization of exosomes. Our protein content analysis indicated that the optimal condition was storage at 4 °C with the preservative, TMO, and that this storage could be maintained for at least 4 weeks. Our study provides relatively comprehensive information on how storage conditions affect EVs. This could inform efforts to better preserve LEVs and significantly contribute to improving the functions and future applications of LEVs.

## 4. Conclusions

We herein investigated the stability of plant leaf-derived extracellular vesicles when stored at various temperatures with and without two selected preservatives. We found that LEVs-TMO were more stable in storage (as reflected by pH, size distribution, etc.), compared to LEVs and LEVs-1,3-BG. We found that the sizes of LEVs-TMO remained between 30 and 200 nm from 0 to 4 weeks and exhibited a nearly unimodal distribution. The zeta potential (a measure of surface charge) of the EVs was influenced by temperature. When stored at 4 °C with TMO (LEVs-TMO), the vesicles maintained their protein contents to a higher degree over 4 weeks, compared to the other tested conditions. Our data suggest that plant leaf-derived extracellular vesicles stored with the preservative, TMO, at 4 °C showed the best stability among the tested formulations and temperatures. However, increasing the efficiency of Evs’ isolation excluding free proteins is still a challenging issue. Additionally, we presume that aggregation between LEVs occurred by 1,3-BG, but the effect of 1,3-BG on the size of EVs should be studied in the future.

Freeze-thaw cycle testing is an important stability test that assesses whether a formula will remain stable under various temperatures. Here, we found that the size distribution of LEVs was affected by increasing cycles of freezing and thawing, taking on a multimodal distribution. We also assessed whether freeze-thawing and various storage conditions influenced the cellular uptake of LEVs. Indeed, 0 cycles of LEVs showed significantly less efficient autologous cellular uptake compared to 1, 3 cycles of LEVs. In contrast, the cellular uptake of LEVs-TMO was relatively stable despite increasing storage time. We hypothesized that LEVs-TMO were more readily internalized due to their acidic pH. However, additional work is needed to test this hypothesis and better understand the impact of pH on cellular internalization.

EVs research can potentially open many new possibilities in the fields of medicine, cosmetics, and nutrition. Together, the results of optimization of storage condition according to preservatives and various condition described herein improve our understanding of the stability of plant-derived EVs by physical properties. Our studies provide critical evidence for how plant-derived EVs can be stored to best preserved. Because we previously confirmed the anti-melanogenic effects, our findings may enable LEVs to be developed for the wide application as the cosmeceutical formulations as well as their nutrition and pharmaceutical product.

## Supplementary Materials

The following are available online at https://www.mdpi.com/article/10.3390/pharmaceutics14020457/s1, Figure S1: Optical microscopy images of mixture of LEVs and 1,3-BG using at 45℃ during 4 weeks. Figure S2: pH tests of LEVs-1,3BG over time. Figure S3: PDI values of LEVs, LEVs-1,3BG, and LEVs-TMO. Figure S4: Size distribution of LEVs-1,3BG over time under different temperature. Figure S5: Zeta potentials over time under different temperatures. Figure S6: Cellular uptake for LEVs under different temperatures. Table S1: Physical characteristics of LEVs. Table S2: Physical characteristics of mixture of LEVs and 1,3 BG. Table S3: Physical characteristics of mixture of LEVs and TMO.

## References

[1] Kim K., Park J., Jung J.-H., Lee R., Park J.-H., Yuk J.M., Hwang H., Yeon J.H. (2021). Cyclic tangential flow filtration system for isolation of extracellular vesicles. APL Bioeng..

[2] Yeon J.H., Jeong H.E., Seo H., Cho S., Kim K., Na D., Chung S., Park J., Choi N., Kang J.Y. (2018). Cancer-derived exosomes trigger endothelial to mesenchymal transition followed by the induction of cancer-associated fibroblasts. Acta Biomater..

[3] Kim J., Li S., Zhang S., Wang J. (2021). Plant-derived Exosome-like Nanoparticles and their Therapeutic Activities. Asian J. Pharm. Sci..

[4] You J.Y., Kang S.J., Rhee W.J. (2021). Isolation of cabbage exosome-like nanovesicles and investigation of their biological activities in human cells. Bioact. Mater..

[5] Lee R., Ko H.J., Kim K., Sohn Y., Min S.Y., Kim J.A., Na D., Yeon J.H. (2020). Anti-melanogenic effects of extracellular vesicles derived from plant leaves and stems in mouse melanoma cells and human healthy skin. J. Extracell. Vesicles.

[6] Reiner A.T., Somoza V. (2019). Extracellular vesicles as vehicles for the delivery of food bioactives. J. Agric. Food Chem..

[7] Pérez-Bermúdez P., Blesa J., Soriano J.M., Marcilla A. (2017). Extracellular vesicles in food: Experimental evidence of their secretion in grape fruits. Eur. J. Pharm. Sci..

[8] Xiong M., Zhang Q., Hu W., Zhao C., Lv W., Yi Y., Wang Y., Tang H., Wu M., Wu Y. (2021). The novel mechanisms and applications of exosomes in dermatology and cutaneous medical aesthetics. Pharmacol. Res..

[9] Kim H., Lee J.W., Han G., Kim K., Yang Y., Kim S.H. (2021). Extracellular vesicles as potential theranostic platforms for skin diseases and aging. Pharmaceutics.

[10] Cho J.H., Hong Y.D., Kim D., Park S.J., Kim J.S., Kim H.-M., Yoon E.J., Cho J.-S. (2022). Confirmation of plant-derived exosomes as bioactive substances for skin application through comparative analysis of keratinocyte transcriptome. Appl. Biol. Chem..

[11] Cho E.-G., Choi S.-Y., Kim H., Choi E.-J., Lee E.-J., Park P.-J., Ko J., Kim K.P., Baek H.S. (2021). Panax ginseng-derived extracellular vesicles facilitate anti-senescence effects in human skin cells: An eco-friendly and sustainable way to use ginseng substances. Cells.

[12] Lee M., Ban J.-J., Im W., Kim M. (2016). Influence of storage condition on exosome recovery. Biotechnol. Bioprocess Eng..

[13] Zhang Y., Bi J., Huang J., Tang Y., Du S., Li P. (2020). Exosome: A review of its classification, isolation techniques, storage, diagnostic and targeted therapy applications. Int. J. Nanomed..

[14] Akuma P., Okagu O.D., Udenigwe C.C. (2019). Naturally occurring exosome vesicles as potential delivery vehicle for bioactive compounds. Front. Sustain. Food Syst..

[15] Kirkbride L., Humphries L., Kozielska P., Curtis H. (2021). Designing a Suitable Stability Protocol in the Face of a Changing Retail Landscape. Cosmetics.

[16] Bahr M.M., Amer M.S., Abo-El-Sooud K., Abdallah A.N., El-Tookhy O.S. (2020). Preservation techniques of stem cells extracellular vesicles: A gate for manufacturing of clinical grade therapeutic extracellular vesicles and long-term clinical trials. Int. J. Vet..

[17] Neupane Y.R., Huang C., Wang X., Chng W.H., Venkatesan G., Zharkova O., Wacker M.G., Czarny B., Storm G., Wang J.-W. (2021). Lyophilization Preserves the Intrinsic Cardioprotective Activity of Bioinspired Cell-Derived Nanovesicles. Pharmaceutics.

[18] Kusuma G.D., Barabadi M., Tan J.L., Morton D.A., Frith J.E., Lim R. (2018). To protect and to preserve: Novel preservation strategies for extracellular vesicles. Front. Pharmacol..

[19] Jeyaram A., Jay S.M. (2018). Preservation and storage stability of extracellular vesicles for therapeutic applications. AAPS J..

[20] Dao H., Lakhani P., Police A., Kallakunta V., Ajjarapu S.S., Wu K.-W., Ponkshe P., Repka M.A., Murthy S.N. (2018). Microbial stability of pharmaceutical and cosmetic products. Aaps Pharmsci. Tech..

[21] Naufalin R. (2019). Natural preservation opportunities and challenges in improving food safety. AIP Conf. Proc..

[22] Suh J.Y., Yun M.E., Lee Y.S., Xuan S.H., Park D.S., Park S.N. (2018). Preservative Efficacies according to the Composition of 1, 3-Butylene Glycol and Alkane Diols in Cosmetics. J. Soc. Cosmet. Sci. Korea.

[23] Mary Ann Liebert, Inc. (1984). Final report on the safety assessment of methylparaben, ethylparaben, propylparaben, and butylparaben. J. Med. Toxicol..

[24] Kinnunen T., Koskela M. (1991). Antibacterial and antifungal properties of propylene glycol, hexylene glycol, and 1, 3-butylene glycol in vitro. Acta Derm. Venereol..

[25] Aly S.E., Sabry B.A., Shaheen M.S., Hathout A.S. (2016). Assessment of antimycotoxigenic and antioxidant activity of star anise (Illicium verum) in vitro. J. Saudi Soc. Agric. Sci..

[26] Yang C.-H., Chang F.-R., Chang H.-W., Wang S.-M., Hsieh M.-C., Chuang L.-Y. (2012). Investigation of the antioxidant activity of Illicium verum extracts. J. Med. Plants Res..

[27] Ahmad A.F., Youssef M.S. (2015). Chemical composition and bioactive properties of Illicium verum (star-anise) extracts prepared by different methods. JCBPS.

[28] Paul R., Geetha R. (2018). Evaluation of anti-inflammatory action of Illicium verum-An in vitro study. Drug Invent. Today.

[29] Fang B., Yu M., Zhang W., Wang F. (2016). A new alternative to cosmetics preservation and the effect of the particle size of the emulsion droplets on preservation efficacy. Int. J. Cosmet. Sci..

[30] Levy S.B., Dulichan A.M., Helman M. (2009). Safety of a preservative system containing 1, 2-hexanediol and caprylyl glycol. Cutan. Ocul. Toxicol..

[31] Song U., Kim J. (2020). Assessment of the potential risk of 1, 2-hexanediol using phytotoxicity and cytotoxicity testing. Ecotoxicol. Environ. Saf..

[32] Hwang S., Park S., Hwang J.K., Pan J.G. (2015). Food-grade antimicrobials potentiate the antibacterial activity of 1, 2-hexanediol. Lett. Appl. Microbiol..

[33] Kim K., Yoo H.J., Jung J.-H., Lee R., Hyun J.-K., Park J.-H., Na D., Yeon J.H. (2020). Cytotoxic effects of plant sap-derived extracellular vesicles on various tumor cell types. J. Funct. Biomater..

[34] Kim K., Jung J.-H., Yoo H.J., Hyun J.-K., Park J.-H., Na D., Yeon J.H. (2020). Anti-Metastatic Effects of Plant Sap-Derived Extracellular Vesicles in a 3D Microfluidic Cancer Metastasis Model. J. Funct. Biomater..

[35] Youn J.S., Kim Y.-J., Na H.J., Jung H.R., Song C.K., Kang S.Y., Kim J.Y. (2019). Antioxidant activity and contents of leaf extracts obtained from Dendropanax morbifera LEV are dependent on the collecting season and extraction conditions. Food Sci. Biotechnol..

[36] Moon H.-I. (2011). Antidiabetic effects of dendropanoxide from leaves of Dendropanax morbifera Leveille in normal and streptozotocin-induced diabetic rats. Hum. Exp. Toxicol..

[37] Choo G.S., Lim D.P., Kim S.M., Yoo E.S., Kim S.H., Kim C.H., Woo J.S., Kim H.J., Jung J.Y. (2019). Anti-inflammatory effects of Dendropanax morbifera in lipopolysaccharide-stimulated RAW264. 7 macrophages and in an animal model of atopic dermatitis. Mol. Med..

[38] Halla N., Fernandes I.P., Heleno S.A., Costa P., Boucherit-Otmani Z., Boucherit K., Rodrigues A.E., Ferreira I.C., Barreiro M.F. (2018). Cosmetics preservation: A review on present strategies. Molecules.

[39] Huang Y., Wang S., Cai Q., Jin H. (2021). Effective methods for isolation and purification of extracellular vesicles from plants. J. Integr. Plant Biol..

[40] Urzì O., Raimondo S., Alessandro R. (2021). Extracellular vesicles from plants: Current knowledge and open questions. Int. J. Mol. Sci..

[41] Rutter B.D., Rutter K.L., Innes R.W. (2017). Isolation and quantification of plant extracellular vesicles. Bio Protoc..

[42] Rodrigues F., Gaspar C., Palmeira-de-Oliveira A., Sarmento B., Helena Amaral M., Oliveira M.B.P.P. (2016). Application of coffee silverskin in cosmetic formulations: Physical/antioxidant stability studies and cytotoxicity effects. Drug Dev. Ind. Pharm..

[43] Fernandes A.R., Dario M.F., Pinto C.A.S.d.O., Kaneko T.M., Baby A.R., Velasco M.V.R. (2013). Stability evaluation of organic Lip Balm. Braz. J. Pharm. Sci..

[44] Beit-Yannai E., Tabak S., Stamer W.D. (2018). Physical exosome: Exosome interactions. J. Cell. Mol. Med..

[45] Amaro-Gahete J., Benítez A., Otero R., Esquivel D., Jiménez-Sanchidrián C., Morales J., Caballero Á., Romero-Salguero F.J. (2019). A comparative study of particle size distribution of graphene nanosheets synthesized by an ultrasound-assisted method. Nanomaterials.

[46] Midekessa G., Godakumara K., Ord J., Viil J., Lättekivi F., Dissanayake K., Kopanchuk S., Rinken A., Andronowska A., Bhattacharjee S. (2020). Zeta potential of extracellular vesicles: Toward understanding the attributes that determine colloidal stability. Acs Omega.

[47] Cheng Y., Zeng Q., Han Q., Xia W. (2019). Effect of pH, temperature and freezing-thawing on quantity changes and cellular uptake of exosomes. Protein Cell.

[48] Sokolova V., Ludwig A.-K., Hornung S., Rotan O., Horn P.A., Epple M., Giebel B. (2011). Characterisation of exosomes derived from human cells by nanoparticle tracking analysis and scanning electron microscopy. Colloids Surf. B Biointerfaces.

[49] Burnouf T., Agrahari V., Agrahari V. (2019). Extracellular vesicles as nanomedicine: Hopes and hurdles in clinical translation. Int. J. Nanomedicine.

[50] Lőrincz Á.M., Timár C.I., Marosvári K.A., Veres D.S., Otrokocsi L., Kittel Á., Ligeti E. (2014). Effect of storage on physical and functional properties of extracellular vesicles derived from neutrophilic granulocytes. J. Extracell. Vesicles.

[51] Maroto R., Zhao Y., Jamaluddin M., Popov V.L., Wang H., Kalubowilage M., Zhang Y., Luisi J., Sun H., Culbertson C.T. (2017). Effects of storage temperature on airway exosome integrity for diagnostic and functional analyses. J. Extracell. Vesicles.

[52] Wu Y., Deng W., Klinke D.J. (2015). Exosomes: Improved methods to characterize their morphology, RNA content, and surface protein biomarkers. Analyst.

[53] Wu J.-Y., Li Y.-J., Hu X.-B., Huang S., Xiang D.-X. (2021). Preservation of small extracellular vesicles for functional analysis and therapeutic applications: A comparative evaluation of storage conditions. Drug Deliv..

[54] Foroozandeh P., Aziz A.A. (2018). Insight into cellular uptake and intracellular trafficking of nanoparticles. Nanoscale Res. Lett..

[55] Parolini I., Federici C., Raggi C., Lugini L., Palleschi S., de Milito A., Coscia C., Iessi E., Logozzi M., Molinari A. (2009). Microenvironmental pH is a key factor for exosome traffic in tumor cells. J. Biol. Chem..

