# Supplementary material for: Stability of Plant Leaf-Derived Extracellular Vesicles According to Preservative and Storage Temperature
Source: Pharmaceutics. 2022 Feb 21;14(2):457. doi: 10.3390/pharmaceutics14020457 (PMC8879201; doi:10.3390/pharmaceutics14020457)
Supplement: Supplementary file 1 [file pharmaceutics-14-00457-s001.zip › pharmaceutics-1560416-supplementary.pdf]

Supplementary data for

Storage Stability of Plant Leaf-Derived Extracellular Vesicles According to Preservative and Storage Temperature

Kimin Kim [1], Jungjae Park [2], Yehjoo Sohn [1], Chan-Eui Oh [1], Ji-Ho Park [3], Jong-Min Yuk [2] and Ju-Hun Yeon [1,*]

[1] Department of Integrative Biosciences, University of Brain Education, Cheonan 31228, Korea; kimini1127@naver.com (K.K.); spielian@naver.com (Y.S.); 97rhdaud@naver.com (C.-E.O.)

[2] Department of Materials Science and Engineering, Korea Advanced Institute of Science and Technology, Daejeon 34141, Korea; jungjae10@kaist.ac.kr (J.P.); jongmin.yuk@kaist.ac.kr (J.-M.Y.)

[3] Department of Bio and Brain engineering, Korea Advanced Institute of Science and Technology, Daejeon 34141, Korea; jihopark@kaist.ac.kr

* Correspondence: jhyeon@ube.ac.kr; Tel.: +82-41-529-2621; Fax: +82-41-529-2674

	Temperature		-20 ℃			4 ℃			25 ℃			45 ℃		
Condition	Date	N	Odor	Color	Phase Separation	Odor	Color	Phase Separation	Odor	Color	Phase Separation	Odor	Color	Phase Separation
LEVs	0 w	3	Slightly characteristic odor	Light yellow	No change	Slightly characteristic odor	Light yellow	No change	Slightly characteristic odor	Light yellow	No change	Slightly characteristic odor	Light yellow	No change
	1 w	3	N.C	N.C	N.C	N.C	N.C	N.C	N.C	N.C	N.C	N.C	N.C	N.C
	2 w	3	N.C	N.C	N.C	N.C	N.C	N.C	N.C	N.C	N.C	N.C	N.C	N.C
	3 w	3	N.C	N.C	N.C	N.C	N.C	N.C	N.C	N.C	N.C	N.C	N.C	N.C
	4 w	3	N.C	N.C	N.C	N.C	N.C	N.C	N.C	N.C	N.C	N.C	N.C	N.C
	5 w	3	N.C	N.C	N.C	N.C	N.C	N.C	N.C	N.C	N.C	N.C	N.C	N.C

Supplementary Table S1. Physical characteristics of LEVs.

	Temperature		-20 ℃			4 ℃			25 ℃			45 ℃		
Condition	Date	N	Odor	Color	Phase Separation	Odor	Color	Phase Separation	Odor	Color	Phase Separation	Odor	Color	Phase Separation
LEVs -1,3BG	0 w	3	Slightly characteristic odor	Light yellow	No change	Slightly characteristic odor	Light yellow	No change	Slightly characteristic odor	Light yellow	No change	Slightly characteristic odor	Light yellow	No change
	1 w	3	N.C	N.C	N.C	N.C	N.C	N.C	N.C	N.C	N.C	N.C	N.C	Soluble sediment
	2 w	3	N.C	N.C	N.C	N.C	N.C	N.C	N.C	N.C	N.C	N.C	N.C	Soluble sediment
	3 w	3	N.C	N.C	N.C	N.C	N.C	N.C	N.C	N.C	N.C	N.C	N.C	Soluble sediment
	4 w	3	N.C	N.C	N.C	N.C	N.C	N.C	N.C	N.C	N.C	N.C	N.C	Soluble sediment
	5 w	3	N.C	N.C	N.C	N.C	N.C	N.C	N.C	N.C	N.C	N.C	N.C	Soluble sediment

Supplementary Table S2. Physical characteristics of mixture of LEVs and 1,3 BG.

	Temperature		-20 ℃			4 ℃			25 ℃			45 ℃		
Condition	Date	N	Odor	Color	Phase Separation	Odor	Color	Phase Separation	Odor	Color	Phase Separation	Odor	Color	Phase Separation
LEVs -TMO	0 w	3	Slightly characteristic odor	Light yellow	No change	Slightly characteristic odor	Light yellow	No change	Slightly characteristic odor	Light yellow	No change	Slightly characteristic odor	Light yellow	No change
	1 w	3	N.C	N.C	N.C	N.C	N.C	N.C	N.C	N.C	N.C	N.C	N.C	N.C
	2 w	3	N.C	N.C	N.C	N.C	N.C	N.C	N.C	N.C	N.C	N.C	N.C	N.C
	3 w	3	N.C	N.C	N.C	N.C	N.C	N.C	N.C	N.C	N.C	N.C	N.C	N.C
	4 w	3	N.C	N.C	N.C	N.C	N.C	N.C	N.C	N.C	N.C	N.C	N.C	N.C
	5 w	3	N.C	N.C	N.C	N.C	N.C	N.C	N.C	N.C	N.C	N.C	N.C	N.C

Supplementary Table S3. Physical characteristics of mixture of LEVs and TMO.

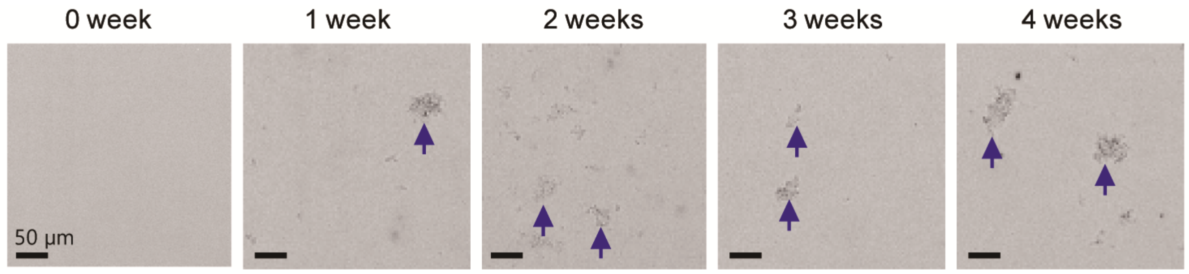

Supplementary Figure S1. Optical microscopy images of mixture of LEVs and 1,3-BG using at 45°C during 4 weeks. The mixture of LEVs and 1,3-BG showed soluble sediment at 45°C from 1 weeks to 4 weeks.

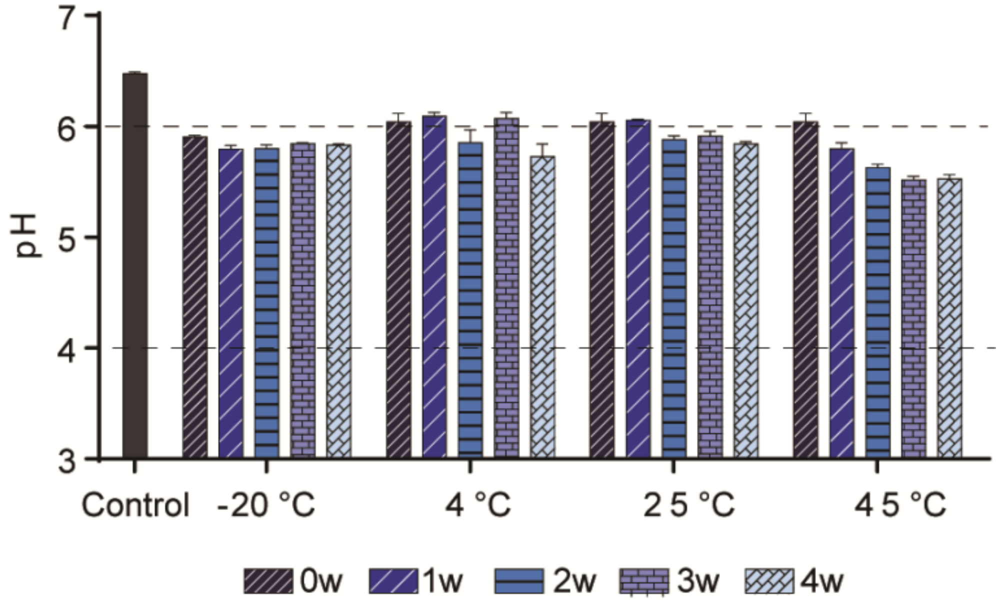

Supplementary Figure S2. pH tests of LEVs-1,3BG over time.

	LEVs	LEVs+1,3BG	LEVs+TMO
PDI	0.238 ± 0.105	0.270 ± 0.045	0.270 ± 0.060

Supplementary Figure S3. PDI values of LEVs, LEVs-1,3BG, and LEVs-TMO.

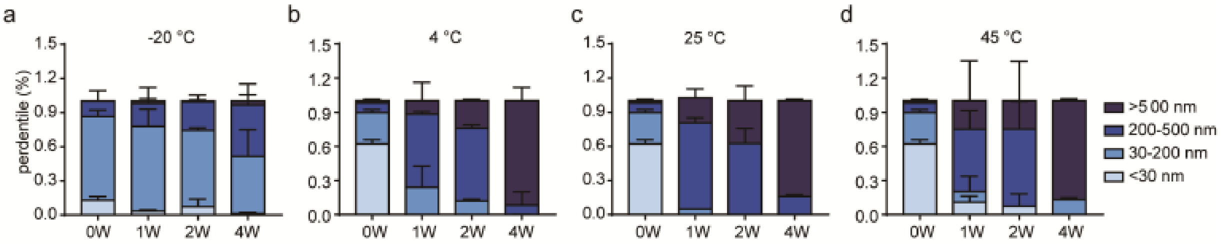

Supplementary Figure S4. Size distribution of LEVs-1,3BG over time under different temperature. The mixture of LEVs and 1,3-BG showed change of size distribution stored at -20, 4, 25, 45 ℃.

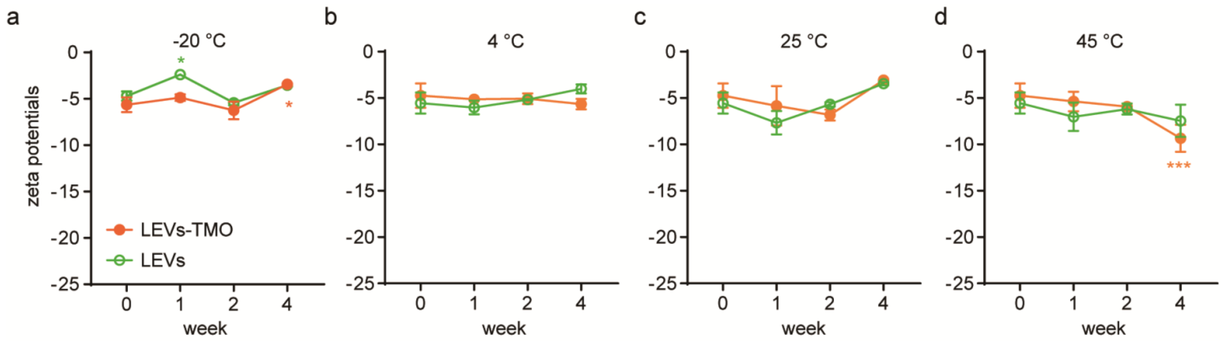

Supplementary Figure S5. Zeta potentials over time under different temperatures. Zeta potentials of LEVs and LEVs-TMO stored at (a) -20, (b) 4, (c) 25, and (d) 45 °C for 4 weeks. Data are presented as mean ± standard error of mean (SEM). (***$p < 0.001$, ****$p < 0.0001$).

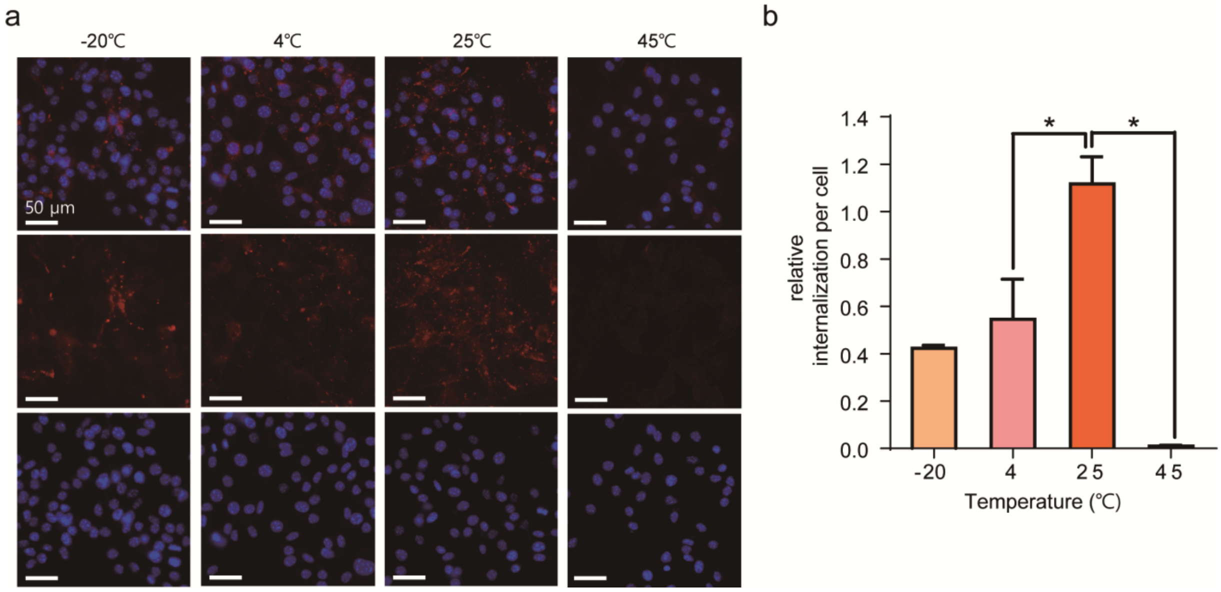

Supplementary Figure S6. Cellular uptake for LEVs under different temperatures. (a) Representative fluorescence microscopic images of cellular uptake for LEVs stored at -20, 4, 25, and 45 °C. (b) Summary data comparing intracellular fluorescence intensity per cell. Data are presented as mean ± standard error of mean (SEM). (*$p < 0.01$).
